# Supplementary material for: New Findings on LMO7 Transcripts, Proteins and Regulatory Regions in Human and Vertebrate Model Organisms and the Intracellular Distribution in Skeletal Muscle Cells
Source: Int J Mol Sci. 2021 Nov 28;22(23):12885. doi: 10.3390/ijms222312885 (PMC8657913; doi:10.3390/ijms222312885)
Supplement: Supplementary file 1 [file ijms-22-12885-s001.zip › ijms-1452171-supplementary.pdf]

Table S1 - Species analyzed and details about the *Lmo7* ortholog genes identified.

Species	NCBI ID	Ensembl ID	Location	Strand	Exons count	UniProt match
Homo sapiens	4008	ENSG00000136153	Ch13q22.2	+	39	Q8WWI1
Mus musculus	380928	ENSMUSG00000033060	Ch14 E2.3	+	37	E9PYF4
Monodelphis domestica	100012301	ENSMODG00000001099	Ch7	+	33	F7DPH4
Gallus gallus	418815	ENSGALG00000016920	Ch1	-	37	F1NY52
Taeniopygia guttata	100225324	ENSTGUG00000012539	Ch1		36	H0ZQM6
Pelodiscus sinensis	102450619	ENSPSIG00000014803	Un	-	32	K7G8Y9
Anolis carolinensis	100561689	ENSACAG00000002156	Ch3	-	34	G1KAN0
Xenopus tropicalis	100216219	ENSXETG00000004710	Ch2		35	F7AC24
Danio rerio	553341 (*lmo7a*)	ENSDARG00000004930	Ch2	-	35	B0UYM8
	558333 (*lmo7b*)	ENSDARG00000053535	Ch1	-	30	F6NN15
Ictalurus punctatus	108266511 (*lmo7a*)	-	Ch6	+	27	A0A2D0R6I3
	108259086 (*lmo7b*)	-	Ch26	-	30	A0A2D0Q7R4
Lepisosteus oculatus	102692305	ENSLOCT00000014072.1	LG17	+	36	W5N084
Branchiostoma floridae	118419103	-	Ch 7	+	39	XP_035681

Table S2 - Genes identified around *Lmo7* ortholog genes in different vertebrate species.

**Species**	**Genes (not all shown)**								
Homo sapiens	**TBC1D4**	**COMMOD6**	**UCHL3**	**LMO7 AS1**	**LMO7**	**FAM204 CP**	**LMO7 DN**	**LINC00561**	
Mus musculus	✓	✓	✓		✓				
Monodelphis domestica	✓	✓	✓		✓				
Gallus gallus	✓	✓	✓		✓				**KCTD12**
Taeniopygia guttata	✓	✓	✓		✓				✓
Pelodiscus sinensis									
Anolis carolinensis	✓	✓	✓		✓				✓
Xenopus tropicalis	✓	✓	✓		✓				✓
Danio rerio			✓		✓ ***=lmo7a***				✓ (kctd12.1)
Ictalurus punctatus			✓		✓				✓
Danio rerio			**Klf12a**		✓ ***=lmo7b***				kctd12.2
Ictalurus punctatus			✓		✓				✓

The ticks represent the gene orthologous to the named gene above.

Table S3 - List of human *Lmo7* predicted alternative transcripts.

Name	Transcript ID	bp	Protein	Translation ID	Biotype	CCDS	UniProt Match	RefSeq Match	Flags
LMO7-201	ENST00000321797.12	5647	1398aa	ENSP00000317802.8	Protein coding	CCDS81774	E9PMT2	-	GENCODE basic, TSL:5,
LMO7-202	ENST00000341547.8	7237	1349aa	ENSP00000342112.4	Protein coding	CCDS9454	**Q8WWI1-3**	-	GENCODE basic, TSL:1,
LMO7-203	ENST00000357063.7	8237	1668aa	ENSP00000349571.4	Protein coding	-	J3KP06	-	GENCODE basic, APPRIS ALT2, TSL:5,
LMO7-204	ENST00000377499.9	3472	1045aa	ENSP00000366719.5	Protein coding	-	E9PMP7	-	TSL:2, CDS 3' incomplete,
LMO7-205	ENST00000377534.8	7147	1631aa	ENSP00000366757.4	Protein coding	CCDS81773	F8WD26	NM_001306080.2	MANE Select v0.93, Ensembl Canonical, GENCODE basic, APPRIS P2, TSL:1,
LMO7-206	ENST00000447038.5	3165	1055aa	ENSP00000388955.1	Protein coding	-	H0Y424	-	TSL:2, CDS 5' and 3' incomplete,
LMO7-207	ENST00000465261.6	5580	1385aa	ENSP00000433352.1	Protein coding	CCDS53876	A0A0A0MTE2	-	GENCODE basic, TSL:1,
LMO7-208	ENST00000465309.6	572	No protein	-	Processed transcript	-	-	-	TSL:3,
LMO7-209	ENST00000467686.1	591	No protein	-	Retained intron	-	-	-	TSL:2,
LMO7-210	ENST00000482116.1	935	No protein	-	Retained intron	-	-	-	TSL:3,
LMO7-211	ENST00000485987.1	1087	No protein	-	Processed transcript	-	-	-	TSL:5,
LMO7-212	ENST00000489941.6	597	165aa	ENSP00000431636.1	Protein coding	-	E9PK58	-	TSL:2, CDS 3' incomplete,
LMO7-213	ENST00000497947.6	995	121aa	ENSP00000431271.1	Protein coding	-	E9PLU6	-	TSL:5, CDS 3' incomplete,
LMO7-214	ENST00000524651.5	914	305aa	ENSP00000433591.1	Protein coding	-	H0YDG6	-	TSL:3, CDS 5' and 3' incomplete,
LMO7-215	ENST00000525107.6	536	179aa	ENSP00000435296.2	Protein coding	-	H0YE95	-	TSL:5, CDS 5' and 3' incomplete,
LMO7-216	ENST00000525373.5	672	191aa	ENSP00000431896.1	Protein coding	-	E9PJ10	-	TSL:4, CDS 3' incomplete,
LMO7-217	ENST00000525914.5	874	292aa	ENSP00000434083.1	Protein coding	-	H0YDQ3	-	TSL:5, CDS 5' and 3' incomplete,
LMO7-218	ENST00000526202.5	4121	1275aa	ENSP00000431129.1	Protein coding	-	E9PMS6	-	GENCODE basic, TSL:2,
LMO7-219	ENST00000526371.1	650	165aa	ENSP00000432269.1	Protein coding	-	E9PRJ0	-	TSL:4, CDS 3' incomplete,
LMO7-220	ENST00000526528.1	572	141aa	ENSP00000434201.1	Protein coding	-	E9PRE3	-	TSL:4, CDS 3' incomplete,
LMO7-221	ENST00000532377.1	595	No protein	-	Retained intron	-	-	-	TSL:3,
LMO7-222	ENST00000532785.1	599	No protein	-	Retained intron	-	-	-	TSL:2,
LMO7-223	ENST00000533299.2	1845	No protein	-	Processed transcript	-	-	-	TSL:3,
LMO7-224	ENST00000533305.5	720	No protein	-	Processed transcript	-	-	-	TSL:5,
LMO7-225	ENST00000533809.2	578	99aa	ENSP00000475811.1	Protein coding	-	U3KQE6	-	TSL:4, CDS 3' incomplete,
LMO7-226	ENST00000534657.5	589	12aa	ENSP00000434943.1	Protein coding	-	E9PMJ6	-	TSL:4, CDS 3' incomplete,
LMO7-227	ENST00000605961.1	2039	No protein	-	Processed transcript	-	-	-	TSL:NA,

The human transcript represented in Figure 2 was highlighted in light pink. The protein predicted to be encoded by this transcript was used in structural analysis presented in this work.

Table S4 - List of mouse *Lmo7* alternative transcripts.

Name	Transcript ID	bp	Protein	Translation ID	Biotype	CCDS	UniProt Match	Flags
Lmo7-201	ENSMUST00000100337.10	6092	1699aa	ENSMUSP00000097910.4	Protein coding	CCDS37001	E9PYF4	GENCODE basic, APPRIS P1, TSL:1,
Lmo7-202	ENSMUST00000159026.8	1986	471aa	ENSMUSP00000124605.2	Protein coding	-	Q3UPW3	TSL:1, CDS 3' incomplete,
Lmo7-203	ENSMUST00000159154.2	3330	No protein	-	Retained intron	-	-	TSL:1,
Lmo7-204	ENSMUST00000159258.8	6006	422aa	ENSMUSP00000125465.2	Nonsense mediated decay	-	E0CZD8	TSL:1,
Lmo7-205	ENSMUST00000159314.8	5774	1453aa	ENSMUSP00000124349.2	Protein coding	CCDS84158	E9PYI7	GENCODE basic, TSL:1,
Lmo7-206	ENSMUST00000159597.8	5423	1604aa	ENSMUSP00000123706.2	Protein coding	-	F6VG99	TSL:5, CDS 5' incomplete,
Lmo7-207	ENSMUST00000159769.8	372	No protein	-	Processed transcript	-	-	TSL:3,
Lmo7-208	ENSMUST00000159797.2	1842	No protein	-	Retained intron	-	-	TSL:1,
Lmo7-209	ENSMUST00000159806.2	3845	1189aa	ENSMUSP00000124300.2	Protein coding	-	F6TFN2	TSL:5, CDS 5' incomplete,
Lmo7-210	ENSMUST00000159812.2	481	No protein	-	Processed transcript	-	-	TSL:3,
Lmo7-211	ENSMUST00000159850.3	960	188aa	ENSMUSP00000159141.2	Protein coding	-	A0A5F8MPW2	TSL:5, CDS 5' incomplete,
Lmo7-212	ENSMUST00000159948.8	2692	No protein	-	Retained intron	-	-	TSL:1,
Lmo7-213	ENSMUST00000160038.2	597	No protein	-	Processed transcript	-	-	TSL:3,
Lmo7-214	ENSMUST00000160876.2	664	No protein	-	Retained intron	-	-	TSL:3,
Lmo7-215	ENSMUST00000161668.8	767	No protein	-	Processed transcript	-	-	TSL:3,
Lmo7-216	ENSMUST00000162091.8	2207	No protein	-	Retained intron	-	-	TSL:1,

The mouse transcript represented in Figure 2 was highlighted in light pink.
The protein predicted to be encoded by this transcript was used in structural analysis presented in this work.

Table S5 - List of chicken Lmo7 predicted alternative transcripts.

Name	Transcript ID	bp	Protein	Translation ID	Biotype	UniProt Match	Flags	
LMO7-201	ENSGALT00000107917.1	4536	1477aa	ENSGALP00000065408.1	Protein coding	A0A3Q2U190	-	
LMO7-202	ENSGALT00000061914.2	4086	1327aa	ENSGALP00000049058.2	Protein coding	A0A1D5P8L0	-	
LMO7-203	ENSGALT00000049862.2	3912	1303aa	ENSGALP00000046352.2	Protein coding	A0A1D5P121	APPRIS P5,	the longest sequence
LMO7-204	ENSGALT00000105784.1	3882	1293aa	ENSGALP00000072051.1	Protein coding	A0A3Q2UKD6	APPRIS ALT2	
LMO7-205	ENSGALT00000053488.2	3741	1246aa	ENSGALP00000047415.2	Protein coding	A0A1D5P409	-	
LMO7-206	ENSGALT00000049796.2	4921	1550aa	ENSGALP00000049909.2	Protein coding	A0A1D5PAZ2	-	
LMO7-207	ENSGALT00000027355.6	4827	1608aa	ENSGALP00000027304.6	Protein coding	F1NY52	-	
LMO7-208	ENSGALT00000067771.2	4638	1545aa	ENSGALP00000055082.1	Protein coding	A0A1D5PQC0	-	

The chichen transcript represented in Figure 2 was highlighted in light pink.
The protein predicted to be encoded by this transcript was used in structural analysis presented in this work.

Table S6 - List of *Xenopus tropicalis Lmo7* predicted alternative transcripts.

Name	Transcript ID	bp	Protein	Translation ID	Biotype	UniProt Match	Flags
lmo7-201	ENSXETT00000076894.1	4368	1215aa	ENSXETP00000081744.1	Protein coding	A0A5G3HMA5	-
lmo7-202	ENSXETT00000010221.4	4485	1254aa	ENSXETP00000010221.4	Protein coding	F7AC24	-
lmo7-203	ENSXETT00000041498.4	4620	1299aa	ENSXETP00000041498.4	Protein coding	F6W2B6	-
lmo7-204	ENSXETT00000099052.1	2109	564aa	ENSXETP00000074891.1	Protein coding	A0A5G3JBH3	-
lmo7-206	ENSXETT00000068604.1	1702	230aa	ENSXETP00000094223.1	Protein coding	Q28GY5	-
lmo7-205	ENSXETT00000010224.4	965	213aa	ENSXETP00000010224.4	Protein coding	F7AC15	-

The *Xenopus* transcript represented in Figure 2 was highlighted in light pink.
The protein predicted to be encoded by this transcript was used in structural analysis presented in this work.

Table S7 - List of zebrafish *lmo7a* and *lmo7b* predicted alternative transcripts.

Zebrafish lmo7a

Name	Transcript ID	bp	Protein	Biotype	UniProt Match	Flags
**lmo7a-201**	ENSDART00000009920.11	6323	1826aa	Protein coding	B0UYM8	APPRIS P1
lmo7a-204	ENSDART00000134768.2	1052	351aa	Protein coding	F1Q7Z5	CDS 5' and 3' incomplete,
lmo7a-202	ENSDART00000090782.6	812	270aa	Protein coding	B0UYM5	CDS 5' and 3' incomplete,
lmo7a-212	ENSDART00000145576.2	754	252aa	Protein coding	F1QL17	CDS 5' and 3' incomplete,
lmo7a-210	ENSDART00000144843.2	663	221aa	Protein coding	F1QJN0	CDS 5' and 3' incomplete,
lmo7a-203	ENSDART00000134084.2	625	46aa	Nonsense mediated	E9QJM8	-
lmo7a-213	ENSDART00000146528.3	962	No	Processed transcript	-	-
lmo7a-209	ENSDART00000143305.3	853	No	Processed transcript	-	-
lmo7a-205	ENSDART00000135395.2	740	No	Processed transcript	-	-
lmo7a-207	ENSDART00000136534.3	733	No	Processed transcript	-	-
lmo7a-211	ENSDART00000145085.2	914	No	Retained intron	-	-
lmo7a-208	ENSDART00000139614.2	895	No	Retained intron	-	-
lmo7a-206	ENSDART00000136349.2	824	No	Retained intron	-	-

Zebrafish

Name	Transcript ID	bp	Protein	Translation ID	Biotype	UniProt Match	Flags
lmo7b-204	ENSDART00000140515.5	6086	1384aa	ENSDARP000001156	Protein coding	F6NN15	APPRIS P1,
lmo7b-205	ENSDART00000143048.4	3975	1173aa	ENSDARP000001192	Protein coding	F1R7D1	CDS 5' incomplete,
lmo7b-201	ENSDART00000083736.4	923	161aa	ENSDARP000000781	Protein coding	Q0P4F5	-
lmo7b-202	ENSDART00000132042.2	541	No	-	Processed	-	-
lmo7b-203	ENSDART00000133543.2	541	No	-	Retained	-	-

The zebrafish transcripts represented in Figure 2 were highlighted in light pink.
The proteins predicted to be encoded by these transcript were used in structural analysis presented in this work.
